# Caloric Restriction Mitigates Kidney Fibrosis in an Aged and Obese Rat Model

**DOI:** 10.3390/antiox12091778

**Published:** 2023-09-18

**Authors:** Daniele La Russa, Laura Barberio, Alessandro Marrone, Anna Perri, Daniela Pellegrino

**Affiliations:** 1 Department of Biology, Ecology and Earth Sciences, University of Calabria, 87036 Rende, Italy; laura.barberio@unical.it (L.B.); alessandro.marrone@unical.it (A.M.); danielapellegrino@unical.it (D.P.); 2 LARSO (Analysis and Research on Oxidative Stress Laboratory), University of Calabria, 87036 Rende, Italy; 3 Department of Experimental and Clinical Medicine, Magna Graecia University, 88100 Catanzaro, Italy; anna.perri@unicz.it

**Keywords:** caloric restriction, renal fibrosis, mesenchymal transition, inflammation, oxidative balance, apoptotic pathways

## Abstract

Caloric restriction is an effective intervention to protract healthspan and lifespan in several animal models from yeast to primates, including humans. Caloric restriction has been found to induce cardiometabolic adaptations associated with improved health and to delay the onset and progression of kidney disease in different species, particularly in rodent models. In both aging and obesity, fibrosis is a hallmark of kidney disease, and epithelial–mesenchymal transition is a key process that leads to fibrosis and renal dysfunction during aging. In this study, we used an aged and obese rat model to evaluate the effect of long-term (6 months) caloric restriction (−40%) on renal damage both from a structural and functional point of view. Renal interstitial fibrosis was analyzed by histological techniques, whereas effects on mesenchymal (N-cadherin, Vimentin, Desmin and α-SMA), antioxidant (SOD1, SOD2, Catalase and GSTP1) inflammatory (YM1 and iNOS) markers and apoptotic/cell cycle (BAX, BCL2, pJNK, Caspase 3 and p27) pathways were investigated using Western blot analysis. Our results clearly showed that caloric restriction promotes cell cycle division and reduces apoptotic injury and fibrosis phenotype through inflammation attenuation and leukocyte infiltration. In conclusion, we highlight the beneficial effects of caloric restriction to preserve elderly kidney function.

## 1. Introduction

Aging is a physiological process regulated by the interaction between environmental and genetic factors [1]. The term inflammaging describes chronic low-grade systemic inflammation, during the aging process, in the absence of infection, and is an important risk factor for both mortality and morbidity in elderly individuals [2]. Another important factor that contributes to the increase in disability in the elderly, but not limited to, is obesity, which represents a new and urgent challenge for public health [3]. Obesity concurs to enhance the risk to develop cardiovascular diseases, type 2 diabetes mellitus [4], cancer [5], dementia [6] and chronic kidney disease (CKD) [7,8]. It has been suggested that obesity raises metabolic imbalances, reduces life span and also impairs cellular processes in a similar mechanism to aging [9]. All tissues are affected by aging process and the kidney constitute one of major target organs of age-related damage leading to an increased incidence of CKD in the elderly [10]. The aged kidney undergoes clinical, macroscopic, microscopic and functional changes causing renal dysfunction [11]. Among these changes, some of the most significant damage is the result of fibrosis [12]. Renal fibrosis contributes to the continuity and permanent decline in renal function and is the final stage of most chronic diseases. The molecular mechanisms underlying the renal fibrosis process are complex and remain poorly understood [13]. Renal fibrosis is a process that involves the deposition in the walls of the glomerular capillaries, in the interstitial space and around the arterioles, of a pathological matrix that contributes to the functional disappearance of the nephron and the surrounding vascular system [14]. Epithelial–mesenchymal transition (EMT) is an important biological mechanism contributing to kidney fibrosis, in which polarized epithelial cells, which physiologically interact with the basement membrane, undergo to multiple biochemical modifications that allow them to assume a cellular mesenchymal phenotype, including migratory capacity, invasiveness, high resistance to apoptosis and significant increase in the production of extra cellular matrix components [15]. Fibrosis usually arises from chronic inflammatory responses and is mediated by inflammatory cells and fibroblasts that release various inflammatory signals that result in the breakdown of different epithelial layers. Commonly studied epithelial and mesenchymal cell markers include: E-cadherin; cytokeratin; ZO-1; laminin-1; Entactin; Syndecan; Muc-1; Desmoplakin a1; miR200 [12]. Oxidative stress is an important inducer of renal fibrosis [16] and previous works have also demonstrated its direct involvement in cardiovascular risk [17,18], in brain ischemia [19], obesity [20] and the aging process [21] but also its protective role in preconditioning cell adaptative response [22]. Macrophages actively participate in the clearance of apoptotic and necrotic cells to determine damage and matrix remodeling to replace tissues from acute and chronic kidney disease [23,24] indeed they are known for their pathogenic role in renal inflammation and fibrosis. Pro-inflammatory macrophages M1 release large amounts of pro-inflammatory mediators such as reactive oxygen species (ROS), circulating TNF and all mediators that amplify inflammation and induce renal fibrosis by secretion of MMP-9. Conversely anti-inflammatory M2 macrophages suppress inflammation and kidney injury through the secretion of anti-inflammatory cytokines such as TGF-β and IL-10 but can also directly promote renal fibrosis [25]. Calorie restriction (CR), a reduction in food intake that occurs without causing malnutrition, represents an alternative to age-related oxidative stress and inflammation. [26,27]. In experimental models, CR involves a diet in which there is a reduction of about 40% of calories over the entire life span of the animal, which translates into a 30–40% increase in the maximum life span [28]. Aside from slowing down the rate of aging, long-term calorie restriction offers multiple health benefits. It prevents, for example, many of the age-related diseases, such as cancer or cardiovascular diseases, which are the leading cause of death in the population and reduces the rate of renal senescence in rats by increasing autophagy [29]. Short-term CR, on the other hand, may produce some of the same effects on longevity and physiological function as long-term caloric restriction in rodents [30,31]; moreover, CR has been shown to improve the balance between oxidative stress and inflammation in both plasma and adipose tissue. [26]. Based on these findings from the literature, the aim of our research is to verify the effectiveness of CR on kidney fibrosis in an aged obese rat model in order to determine the mechanisms underlying EMT and fibrosis, as well as to validate the use of the elderly/obese animal model as a phenocopy of systemic aging.

## 2. Materials and Methods

*Animals.* Experimental procedures were performed on young (Y, 15–17 weeks old, *n* = 6) and aged (72 weeks old, *n* = 12) male Sprague–Dawley rats. Animals were individually housed in the animal care facility of the University of Calabria (Italy) under controlled light (12 h light/dark cycle) and temperature (23–25 °C) conditions and with free access to food and water. The old animals were then divided into two subgroups: control rats (OA), which continued to follow an ad libitum diet of a standard laboratory meal (diet ssnif V1535, German; metabolizable energy 3.057 Kcal/kg), and the food restriction rats (CRA), which were fed a diet of the same chow, restricted to 60% of the intake measured by weight in paired control chow-fed rats. The food restriction diet was continued for a total period of 6 months, then aged animals (OA-control and CRA-treated) were sacrificed at 24 months of age. Water and food intakes were recorded every other day, while body mass was recorded monthly. At the end of in vivo experimental protocols, animals were euthanized with isoflurane (4%) followed by cervical transection. The tissues and organs of interest were immediately removed, rapidly rinsed with 150 mM NaCl solution to remove excess blood and then stored at −80 °C until use. Care and use of laboratory animals (Directive 26/2014/EU) were approved by the local ethical committee of the University of Calabria and by the Italian Ministry of Health (license n.295/2016-PR).

*Masson’s trichrome staining.* For histological examination, kidneys samples were fixed in Tissue-Tek^®^ O.C.T., a gel-like compound used to rapidly embed fresh tissue specimens for frozen sections, using a Leica cryostat (CM1950). The slices obtained were then fixed in 4% paraformaldehyde and stained with Masson’s trichrome staining. For the staining with Masson’s trichrome protocol, kidney tissue slides were preheated in a Bouin’s solution at 54–64 °C for 60 min and then washed in running tap water for 10 min. All slides were incubated in Weigert’s haematoxylin for 5 min followed by washing in running tap water for 2 min. The slides were then stained with acid fuchsin for 15 min and then rinsed in distilled water. Next, the slides were treated with phosphomolybdic acid solution for 10 min and then immediately stained with Aniline Blue solution for 5–10 min. Slides were rinsed with distilled water and treated with 1% acetic acid solution for 3–5 min. Each slide was dehydrated through two changes of alcohol and finally soaked in xylene twice. The sections were examined by optical microscope (DM1000 LED; Leica Microsystems, Wetzlar, Germany) and staining quantification of fibrotic areas (stained blue) and cortex areas (stained red) was performed using Image J analysis software 1.52a version, National Institutes of Health, Bethesda, MD, USA.

*Western Blot and Densitometric Analysis.* Kidney samples taken from of each experimental group were rapidly lysed in ice-cold RIPA buffer (Sigma Aldrich, St. Louis, MI, USA) supplemented with a protease inhibitor cocktail (Sigma-Aldrich, Milan, Italy) and then centrifuged at 20,817× *g* for 20 min at 4 °C. Bradford assay (Sigma, St. Louis, MO, USA) was used to evaluate protein concentration in supernatants samples and the same amounts of total protein were separated on sodium dodecyl sulfate polyacrylamide gel electrophoresis (SDS-PAGE gel) and then transferred to Nitrocellulose membrane (NitroBind, Maine Manufacturing, Kennebunk, ME, USA) using a mini transblot (BioRad Laboratories, Hercules, CA, USA). Membranes were then blocked for 1 h at room temperature with 5% non-fat dried milk in 0.05% Tween-20 TRIS-buffered saline (TBS-T) solution and incubated overnight at 4 °C with the following primary antibodies directed against: iNOS (Sigma Aldrich, St. Louis, MI, USA), α-SMA, Bax, Bcl2, Caspase3, Desmin, E-cadherin, GSTP1, N-cadherin, p27, pJNK, SOD1, SOD2, vimentin (Santa Cruz Biotechnology, Inc., Dallas, TX, USA), YM1 (STEMCELL Technologies Canada Inc., Vancouver, BC, Canada) and β-actin (used as loading controls for protein normalization) followed by species-specific peroxidase-linked secondary antibodies (1:2000; Santa Cruz Biotechnology Inc., Dallas, TX, USA) for 1 h at room temperature. Immunodetection was performed with an enhanced chemiluminescence kit (Western Blotting Luminol Reagent, Santa Cruz Biotechnology Inc., Dallas, TX, USA), and the images were captured with the Invitrogen iBright FL1500 Imaging System. Digitalized immunoblots were subjected to densitometric analysis performed using ImageJ software (1.52a version, National Institutes of Health, Bethesda, Rockville, MD, USA).

*Statistical Analysis.* Data were analyzed by one-way analysis of variance (ANOVA), followed by the Bonferroni and Tukey’s multiple comparison test using GraphPad/Prism version 5.01 statistical software (SAS Institute, Abacus Concept Inc., Berkeley, CA, USA). Data are expressed as mean ± standard error (SE).

## 3. Results

### 3.1. Caloric Restriction Decreases Interstitial Collagen Deposition

**Figure 1:**
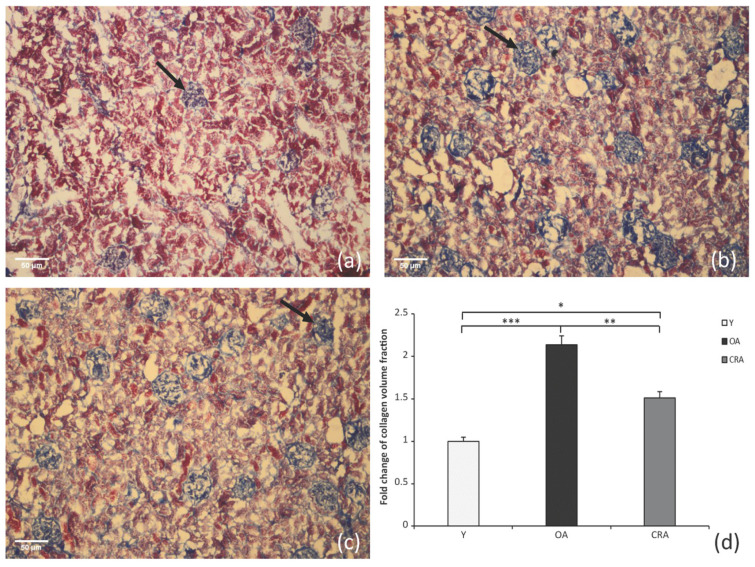
Masson’s trichrome staining of collagen (blue area) and cortex (red area) in kidney sections. Black arrow points to the fibrotic area. Representative staining image from the group Y (**a**), OA (**b**), CRA (**c**); original magnification, ×4. Staining quantification (**d**) by Image-J software 1.52a version, National Institutes of Health, Bethesda, MD, USA (mean ± S.D) are result of 3 animals and 20 casual determinations for each group (* *p* < 0.05; ** *p* < 0.001; *** *p* < 0.0001).

Histological sections of renal samples of Y, OA and CRA were subjected to Masson’s trichrome staining to determine the extracellular matrix components, specifically interstitial and perivascular area collagen deposition. Masson’s staining revealed that the extent of fibrosis in interstitial regions is very high in OA group (Figure 1b) compared to young animals (Figure 1a) and that CR attenuated the collagen accumulated in the renal *interstitium* ameliorating the renal injury (Figure 1c). This result is well evidenced in image collagen quantification in Figure 1d.

### 3.2. Caloric Restriction Downregulates Mesenchymal Markers and Restores E-Cadherin Expression

**Figure 2:**
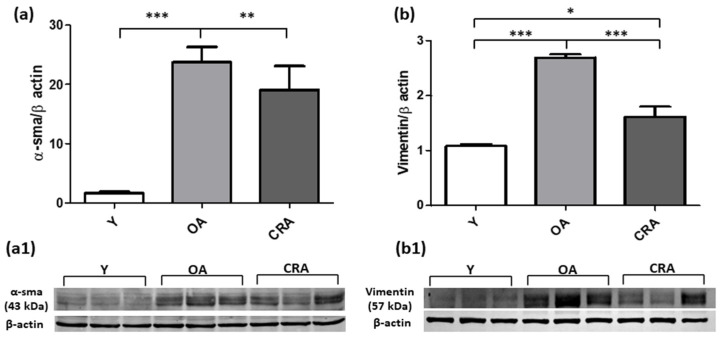
α-SMA (**a**) and Vimentin (**b**) protein expression in kidney tissue samples from Y, OA and CRA rats. (**a1**,**b1**) show the densitometric quantification of the blots. β-actin levels were used as loading control. Data are expressed as means ± SE of five determinations for each animal (*n* = 6). One-way ANOVA followed by Bonferroni’s multiple comparison test were used to analyze statistical differences (* *p* < 0.05; ** *p* < 0.001; *** *p* < 0.0001).

**Figure 3:**
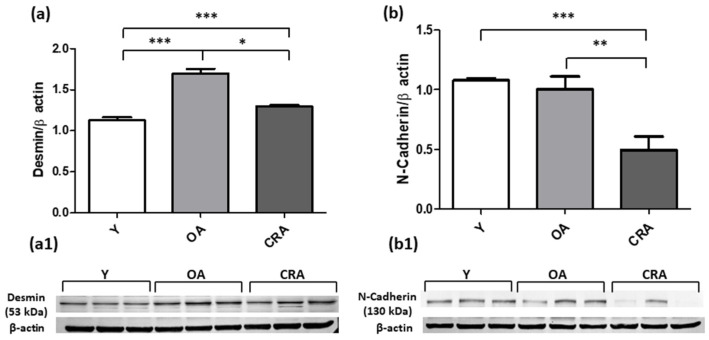
Desmin (**a**) and N-Cadherin (**b**) proteins expression in kidney tissue samples from Y, OA and CRA rats. (**a1**,**b1**) show the densitometric quantification of the blots. β-actin levels were used as loading control. Data are expressed as means ± SE of five determinations for each animal (*n* = 6). One-way ANOVA followed by Bonferroni’s multiple comparison test (* *p* < 0.05; ** *p* < 0.001; *** *p* < 0.0001) was used to analyze statistical differences.

**Figure 4:**
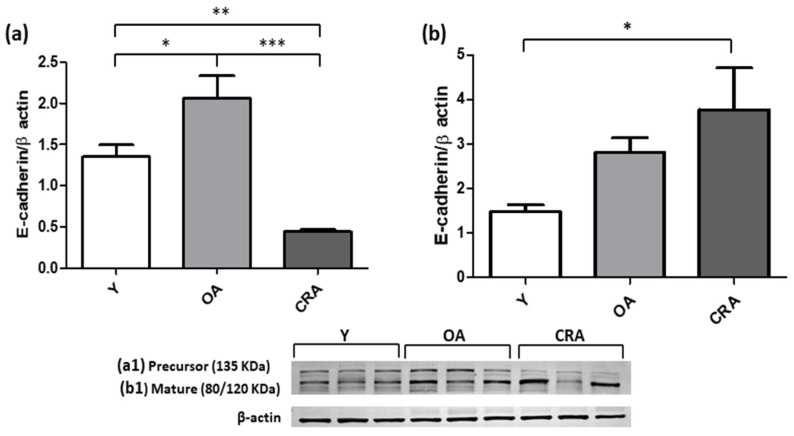
Expression of Precursor (**a**) and Mature (**b**) form of E-cadherin protein in kidney tissue samples of Y, OA and CRA rats. (**a1**,**b1**) show the densitometric quantification of the blots. β-actin level was used as loading control. Data are expressed as means ± SE of five determinations for each animal (*n* = 6). One-way ANOVA followed by Bonferroni’s multiple comparison test (* *p* < 0.05; ** *p* < 0.001; *** *p* < 0.0001) were used to analyze statistical differences.

Western blot analysis was used for detecting the mesenchymal markers α-SMA, Vimentin, Desmin and N-cadherin. The increased α-SMA and Vimentin protein levels in OA animals was significantly reduced in CRA group (Figure 2a,b). A similar expression trend was also observed for Desmin protein, whose protein level was reported to control condition (Figure 3a). Concerning N-cadherin, our results showed that CRA suppressed its protein level expression far below those observed in the other experimental groups (Figure 3b). Interestingly, concerning the epithelial marker E-cadherin, dietary restriction significantly restores the expression of the mature protein form compared to all the experimental groups, but did not affect the precursor protein form that was lower in the caloric restricted rats compared to the other experimental groups (Figure 4a,b).

### 3.3. Caloric Restriction Affects Renal Inflammation

**Figure 5:**
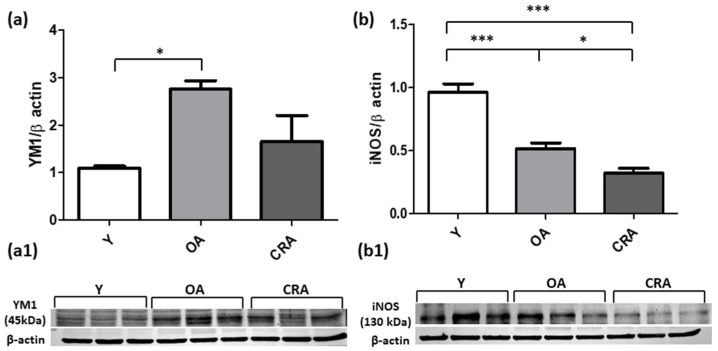
YM1 (**a**) and iNOS (**b**) proteins expression in kidney tissue samples of Y, OA and CRA rats. (**a1**,**b1**) show the densitometric quantification of the blots. β-actin levels were used as loading control. Data are expressed as means ± SE of five determinations for each animal (*n* = 6). One-way ANOVA followed by Bonferroni’s multiple comparison test (* *p* < 0.05; *** *p* < 0.0001) were used to analyze statistical differences.

Given the significance of inflammation in kidney fibrosis, we determined the level expression of YM1 (an M2 macrophage marker) and iNOS (an M1 macrophage marker) proteins. Western blot analysis demonstrated that CR mitigates the M2 macrophage infiltration and suppressed iNOS protein expression compared to both OA and Y rats (Figure 5a,b). These results highlight the potential benefits of diet intervention on renal inflammatory profile which can contribute to mitigate kidney fibrosis.

### 3.4. Caloric Restriction Protects from Renal Apoptotic Injury and Promotes Cell Division

**Figure 6:**
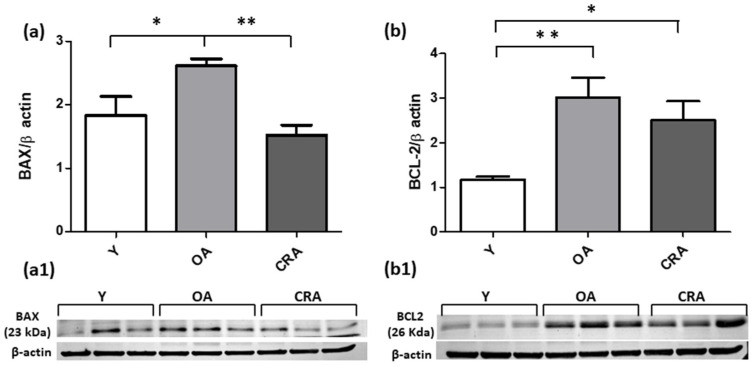
BAX (**a**) and BCL2 (**b**) proteins expression in kidney tissue samples from Y, OA and CRA rats. (**a1**,**b1**) show the densitometric quantification of the blots. β-actin levels were used as loading control. Data are expressed as means ± SE of five determinations for each animal (*n* = 6). One-way ANOVA followed by Bonferroni’s multiple comparison test (Figure (**a1**) * *p* < 0.05; ** *p* < 0.001) and Tukey’s multiple comparison test (Figure (**b1**) * *p* < 0.05; ** *p* < 0.001) were used to analyze statistical differences.

**Figure 7:**
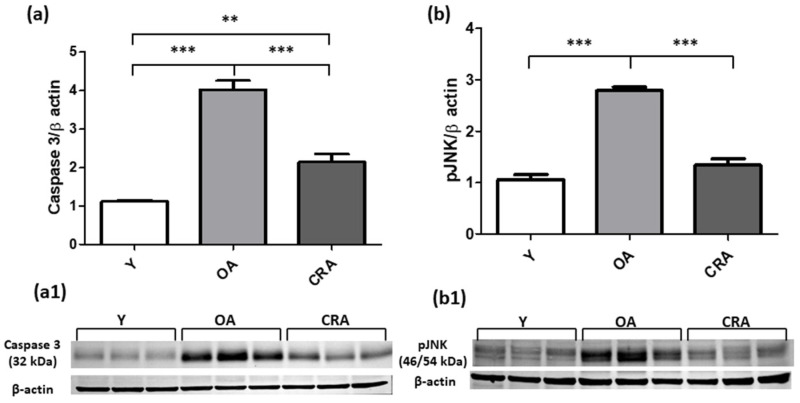
Caspase 3 (**a**) and pJNK (**b**) proteins expression in kidney tissue samples from Y, OA and CRA rats. (**a1**,**b1**) show the densitometric quantification of the blots. β-actin levels were used as loading control. Data are expressed as means ± SE of five determinations for each animal (*n* = 6). One-way ANOVA followed by Bonferroni’s multiple comparison test (** *p* < 0.001, *** *p* < 0.0001) were used to analyze statistical differences.

**Figure 8:**
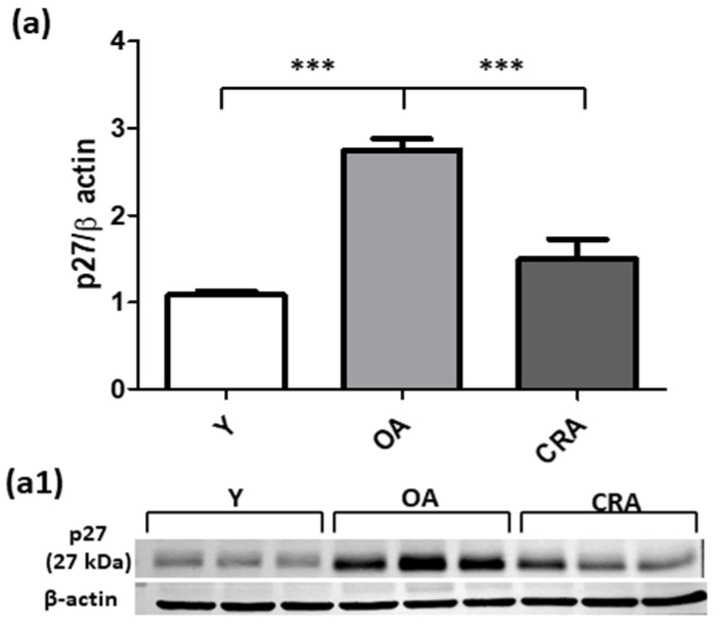
p27 protein expression (**a**) in kidney tissue samples from Y, OA and CRA rats. (**a1**) show the densitometric quantification of the blots. β-actin level was used as loading control. Data are expressed as means ± SE of five determinations for each animal (*n* = 6). One-way ANOVA followed by Bonferroni’s multiple comparison test (*** *p* < 0.0001) were used to analyze statistical differences.

In the setting of organ fibrosis, ongoing inflammation can lead to organ destruction and for this reason we analyzed the apoptotic pathways markers. Our results showed that the follow apoptotic signaling mediators analyzed (Bax, Caspase 3, pJNK) were significantly upregulated by obese and aging conditions and suppressed and returned to control condition by CR (Figure 6a and Figure 7a,b). Concerning Bcl-2 (Figure 6b), we did not detect an increase in its protein level but only a slight decrease in CRA group compared to OA rats. To further confirm the protective role of CR on renal senescence, we highlighted a significant downregulation of the p27 protein, a negative regulator of the cell cycle (Figure 8).

### 3.5. Caloric Restriction Enhances SOD1 Expression but Negatively Modulates GSTP1 Protein Level

**Figure 9:**
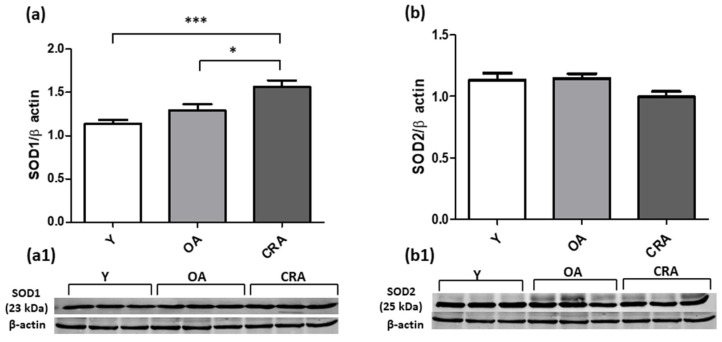
SOD1 (**a**) and SOD2 (**b**) proteins expression in kidney tissue samples from Y, OA and CRA rats. (**a1**,**b1**) show the densitometric quantification of the blots. β-actin levels were used as loading control. Data are expressed as means ± SE of five determinations for each animal (*n* = 6). One-way ANOVA followed by Bonferroni’s multiple comparison test (* *p* < 0.05; *** *p* < 0.0001) were used to analyze statistical differences.

**Figure 10:**
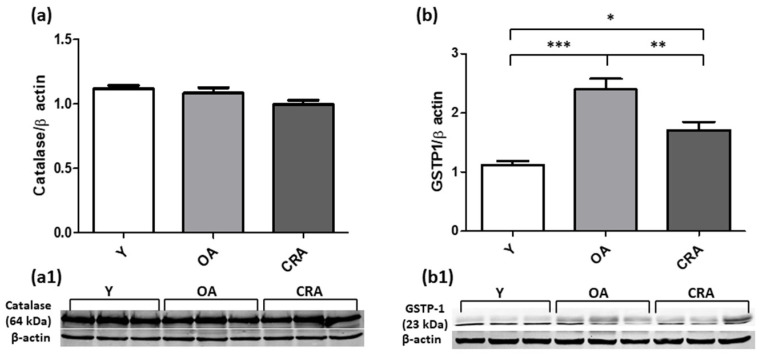
Catalase (**a**) and GSTP-1 (**b**) proteins expression in kidney tissue samples from Y, OA and CRA rats. (**a1**,**b1**) show the densitometric quantification of the blots. β-actin levels were used as loading control. Data are expressed as means ± SE of five determinations for each animal (*n* = 6). One-way ANOVA followed by Bonferroni’s multiple comparison test (* *p* < 0.05; ** *p* < 0.001; *** *p* < 0.0001) were used to analyze statistical differences.

To evaluate the involvement of oxidative stress in the fibrotic process, we analyzed the expression of key antioxidant enzymes. Our results demonstrated that CR enhances the expression of the cytoplasmic antioxidant enzyme SOD1 without affecting the expression of the mitochondrial SOD2 isoform (Figure 9a,b). Concerning GSTP1 antioxidant enzyme, our results showed a notable increase in expression in obese rats and a considerably reduced expression in animals subjected to CR, although it remains high compared to the control group (Figure 10a). Catalase protein expression instead was not affected by CR and remains comparable in all experimental groups (Figure 10b).

## 4. Discussion

Our study was designed to analyze the effect of long-term (6 months) CR (−40%) on the kidney structural and functional damage in an aged and obese rat model. We demonstrated that CR represents an efficient strategy to protect kidney morpho-functional structure in the elderly. Our results clearly showed that CR promotes cell cycle division, reduces apoptotic injury and fibrosis phenotype through the inflammation attenuation and leukocytes infiltration. The experimental model we used proved to be particularly functional for aging studies as it exhibits a gradual and age-related increase in body weight complemented by a phenotypic change in the body’s fat redistribution that affects energy metabolism and systemic insulin resistance [32] with close similarities to aged human [33]. In particular, our aged rats (72 weeks old) present a 45% increased body weight compared to the young animals, and therefore have overt obesity [34] and also show a oxidative imbalance and low grade proinflammatory state typical in both obesity and aging [26,35]. The molecular basis of ageing is not yet well known; however, the functional and structural changes associated with ageing and the ways in which genetic background, age and disease can combine to produce functional damage are becoming increasingly evident. As for all organs, the aging of the kidney involves physiological morpho-functional alterations; however, in the case of this organ, the changes associated with age undergoes complex alterations that predispose to renal dysfunction [36,37]. The renal senescence phenotype is characterized by tissue degeneration and loss of functioning, even though it is unclear whether these changes are primary or secondary events [36]. Replicative senescence and oxidative stress seem to play a fundamental role in the process of renal aging, creating an oxidative hypoxic/ischemic environment complicated by both genetic and environmental factors [38,39]. At structural level, the senescent kidney shows a significant reduction in the overall mass (up to 20–30%) particularly in cortex, and an important interstitial fibrosis [36]. Our results confirm that the condition of obesity associated with aging determines a high level of fibrosis in the renal interstitial regions, but our interesting data is that this condition is partially reversible, as CR significantly mitigates the important interstitial fibrosis occurred in the elderly and obese specimens. It has been shown that CR counteracts all morphologically age-associated changes including interstitial fibrosis, but these protective effects of calorie restriction have been found to be fundamentally preventive [29,40,41,42,43]. Interstitial fibrosis typical of the senescent kidney is associated with EMT [11], a known phenomenon in both physiological and pathological processes, which involves a functional transition of polarized epithelial cells into motile mesenchymal cells that secrete a series of markers including α-SMA, Vimentin, Desmin and N-cadherin [12]. In both human and murine models of senescent kidney, renal epithelial cells seem particularly sensitive to EMT stimuli that occur in response to inflammatory/oxidative stress, indeed in renal fibrosis about 35% of fibroblasts derive from endothelial cells through this complex pathologic process [11,44]. In our aged and obese rat model, we detected high levels of mesenchymal markers and also in this case, the CR has downregulated their expression. However, since most of these mesenchymal markers are not absolutely specific, as they are also present in other cells [45], we also evaluated the epithelial marker E-cadherin, frequently used to characterize the subsistence of EMT and fibrosis in renal tissues [46,47]. Interestingly, dietary restriction significantly restores the expression of the mature E-cadherin form but did not affect the precursor protein form that was lower in the caloric restricted rats compared to the other experimental groups. Although the mature form of E-cadherin protein is involved in the EMT process [48] our results highlight that in caloric restricted rats all the precursor E-cadherin protein form is turned into the mature protein form while this protein activation process is not fully triggered in the other experimental groups. To analyze the putative anti-inflammatory properties of diet intervention on renal fibrosis, we determined the level expression of an M2 macrophage marker, YM1, and the inducible isoform of NOS, iNOS, a hallmark of M1 macrophage. Macrophages are a heterogeneous population with an important role in kidney homeostasis. They play a very complex role in kidney fibrosis [49,50] and their recruitment/activation, is deemed as a key factor behind fibrotic process [25]. After kidney injury, both resident and infiltrating macrophages are rapidly enrolled to the glomerulus or tubulointerstitium to initiate innate immune responses and promote defensive as well as destructive process in kidney tissue [51]. This dualistic role of macrophages is explained by their phenotypic heterogeneity and functional diversity [52]. In rats, in the early stage of kidney ischemia–reperfusion injury (IRI), pro-inflammatory M1 macrophages highly express iNOS [53] a key factor induced by several chronic inflammatory state (type 2 diabetes, cardiovascular disease and hypertension) including kidney disease [54,55] and obesity [56,57]. M1 macrophages secrete a series of pro-inflammatory factors which promote inflammation and tissue damage [58,59]. Many studies demonstrated a protective role of NO following the increase in eNOS and iNOS expression [60,61] but in the context of kidney fibrosis, iNOS may contribute to EMT process [62,63]. This dualistic role of iNOS could explain the differences found in Y, OA and CRA experimental groups: in young rats iNOS protein was significantly upregulated and this protein trend which in our experimental condition such as inflammaging process and obesity may contribute to kidney fibrosis, was decreased by caloric restriction. Alternatively, activated M2 anti-inflammatory macrophages promote both tissue repair [52], but also kidney fibrosis via secretion of TGF-β1 [50]. M2 macrophages, which highly express Arg1, CD206 and chitinase-like proteins such as Ym1 and Fizz1 provide for repairing the affected tissue, but upon chronic injury, they promote renal fibrosis through paracrine effects or direct transition to myofibroblast-like cells, via the process of macrophage-to-myofibroblast transition [64]. According to this evidence, our experiment’s results showed that CR significantly attenuates YM1 expression and mitigates their profibrotic stimulus in kidney tissue. Proximal tubular injury and apoptosis are hallmarks for the development of kidney fibrosis. Injured tubular cells due to an abnormal repair process, undergo to G2/M cell cycle arrest resulting in the secretion of profibrotic cytokines and the activation of a profibrotic signaling pathway [65] thus, the tubule cells assume a senescent secretory phenotype. The inability of adjacent epithelial cells to replace the injured region by proliferation leads to kidney functional loss [66]. Fibrogenic factors released from damaged tubules, such as TGF-β and CTGF, cause the activation of fibroblasts and recruitment of immune cells into the damaged site. Bcl-2 proapoptotic family members, Bcl-2-associated X (BAX) and Bcl-2 antagonist (BAK) are regulators of intrinsic cell apoptosis which mediate outer mitochondrial membrane permeabilization [67]. These proteins are inhibited by pro-survival Bcl-2 proteins and many studies have demonstrated that BAX and BAK proteins participate in apoptotic/necrotic process in kidney disease and the silencing of their functions could prevent apoptosis and the subsequent fibrogenic signaling [68,69]. Our present findings showed that CR inhibit the pro-apoptotic proteins BAX, pJNK and Caspase 3 by enhancing BCL-2 expression. However, this last result is also in according with evidence from the literature that highlight a non-apoptotic role of Bcl-2 related to an inhibitory effect on the cell cycle depending on a cyclin/cyclin-dependent kinase (CDK) inhibitor p27 [70,71,72]. In agreement with these observations our results showed that Bcl-2 overexpression in OA vs Y group, results in elevated levels of p27 protein. This effect was counteracted by CR which promotes cell division and prevents cellular senescence through the downregulation of p27 protein expression. The kidney is also a high energy demand organ, with high levels of oxidation within cellular mitochondria and antioxidant enzymes, such as SOD-1, catalase and GSTP-1, which are the first line cellular antioxidant in many cells. Disturbances in cellular anti-oxidant systems can contribute to renal aging process, cell apoptosis, renal fibrosis and decreased kidney cell renewal [73]. The aging process has been associated with mitochondrial dysfunction, which affects not only the mitochondrial biogenesis process but also decreases the oxidative phosphorylation rate and increases ROS production [74]. Reduced activity of the respiratory chain was described in many tissues such as the following: cardiac and skeletal muscle, liver, brain, kidney, platelets and lung [75,76,77,78,79]. Two major ROS production sites which are believed to be impaired by aging process are complexes I and III [80,81]. ROS overproduction have been suggested as the primary force of age-related cellular damage and for this purpose many theories speculate the putative detrimental effects of ROS, including the free radical and mitochondrial theories of aging [82,83,84,85]. Aging is also accompanied by a decreased activity in antioxidant enzymes [86] and by increases in their protein expression to counteract the damaging effects of ROS [87]. Caloric restriction has a beneficial effect on mitochondrial function and causes a significant reduction of mitochondrial ROS production because it induces the transcription of ROS scavenging genes [88,89,90]. Murine studies which targeted antioxidant enzymes such as mitochondrial Mn-superoxide dismutase (Mn-SOD), SOD1 and catalase have also demonstrated that their manipulation may impact lifespan suggesting a possible correlation between the level of oxidative damage and aging [86,87,88,89,90,91]. In kidney tissue, SOD-1 upregulation attenuates uric acid–kidney fibrosis [92], while its downregulation accelerates the progression of diabetic nephropathy and decreases lifespan and accelerated aging process, especially in the renal tissue [93,94]. CR in our experimental model significantly upregulates SOD-1 expression which in turn counteracts oxidative stress, reduces kidney fibrosis and prevents cellular senescence. The same results were obtained in liver tissue, where CR produced an increase in SOD-1 protein level in aged and restricted diet mice compared to both young or aged control group to mitigate lipid peroxidation [95]. Concerning SOD-2 and Catalase, we did not detect modulationand these results are in line with much of the evidence from the literature, which confirmed that the restriction diet has no relevant effect in both mRNA and protein levels of antioxidant enzymes but instead on their activities, in particular in kidney tissue, the results from which were relatively responsive to restricted diet. In fact, previous works reviewed by Walsh and colleagues [96] reported decreases in kidney GPx and SOD activity and increases in catalase activity, suggesting that catalase modulation may be a response signaling by which CR protects against renal disease because it promotes hydrogen peroxide detoxification. In our experimental model we have not detected modulation in SOD-2 or Catalase protein levels, therefore CR preserves the antioxidant enzymes proteins levels and probably through the modulation of their activity levels it counteracts the oxidative damage resulting from aging and obesity. On the contrary, GSTP-1 expression was negatively regulated by CR and this effect, already observed by our group [26], is probably due to the linkage between GSTP1 expression and apoptotic signaling pathways through the activation of c-Jun N-terminal kinase (JNK) [97]. GSTP1-1 also participates in S-glutathionylation reactions and may function as a S-glutathionylase. This reaction is important in the activation catalytic cycle of Peroxiredoxin VI (Prdx6), a singular catalytic cysteine-containing peroxiredoxin which exhibits both glutathione (GSH) peroxidase and 2 phospholipase A2 enzymatic activities [98], involved in the reduction of H_2_O_2_ and phospholipid hydroperoxides [99]. In the S-glutathionylation/activation reaction of peroxiredoxin, the oxidized monomer of Prdx6 resulting from reduction of phospholipid hydroperoxide (PLPCOOH) or H_2_O_2_, forms a heterodimer with thiolate anion of GSTP. The interaction between S-glutathionylated catalytic Cys47 of Prdx6 and the catalytic Cys47 of GSTP1 causes the formation of a heterodimer structure which exposes the disulfide bound to GSH leading to sequential reduction/activation of both Prdx6 and GSTP catalytic cysteines [100]. In the obese and aged rat model, we observed apoptosis activation JNK-mediated a process in which GSTP1 cellular pool is employed for both antioxidant (monomeric form) and apoptotic (dimeric form) functions. On the contrary CR by reducing the apoptotic rate mitigates GSTP-1 requirement by promoting SOD-1 expression.

## 5. Conclusions

Overall, these results highlight the beneficial effects of CR in preventing age-related kidney fibrosis through the reduction of inflammation, oxidative stress and apoptosis. In addition, CR may represent an efficacy strategy to preserve kidney function in the elderly because prevents cellular senescence and promotes cellular turnover.

## Data Availability

The data presented in this study are available on reasonable request from the corresponding author.
